# A simple and reliable adult uncuffed endotracheal tube for combined forceps and cryoprobe biopsy during bronchoscopy

**DOI:** 10.1111/crj.13539

**Published:** 2022-09-15

**Authors:** Meimei Tao, Xinxia Wang, Qian Sun, Hui Li, Hang Zou, Guangfa Zhu

**Affiliations:** ^1^ Department of Pulmonary and Critical Care Medicine, Beijing Anzhen Hospital Capital Medical University Beijing China; ^2^ Beijing Institute of Heart, Lung and Blood Vessel Diseases Beijing China; ^3^ Department of Pulmonary and Critical Care Medicine, Dongzhimen Hospital Beijing University of Chinese Medicine Beijing China

**Keywords:** anesthesia, arrhythmia, forceps and cryoprobe biopsy, oxygen saturation, spontaneous respiration, uncuffed endotracheal tube

## Abstract

**Introduction:**

Combined forceps and cryoprobe biopsy during bronchoscopy are increasingly used. However, the adult standard cuffed endotracheal tube (SCETT) is can be limited by general anaesthesia and neuromuscular blockade. An adult uncuffed endotracheal tube (UCETT) might provide simple and safe airway support in stable patients during forceps and cryoprobe biopsy under spontaneous respiration.

**Methods:**

A retrospective review of stable patients undergoing forceps and cryoprobe biopsy was performed. They were divided into a UCETT group (*N* = 33) and a SCETT group (*N* = 27). The primary technical outcome was the successful intubation and completion of bronchoscopy. The primary safety outcome was the incidence of desaturation events. Recovery time and side effects were also recorded.

**Results:**

UCETTs and SCETTs were successfully inserted, and bronchoscopic procedures were completed in all patients. Only 3/33 (9.1%) patients in the UCETT group exhibited a drop of SPO_2_ < 90% during the bronchoscopy, compared to 2/27 (7.4%) patients in the SCETT group (*P* = 0.545). Patients recovered faster in the UCETT group than those in the SCETT group. Major bleeding, laryngospasm and major arrhythmias did not occur in either group. Incidences of sinus tachycardia, incidences of vomiting, minor and moderate bleeding and premature atrial contractions were not significantly different between the two groups. Nausea occurred in 5/33 (15.2%) patients in the UCETT group, compared to 11/27 (40.7%) in the SCETT group.

**Conclusion:**

This study suggests that UCETT under spontaneous respiration can provide satisfactory airway support and a shorter recovery time in stable patients; thus, it may be an option to assist forceps and cryoprobe biopsy.

## Graphical abstract

An adult uncuffed endotracheal tube (UCETT) group (*N* = 33) and a standard cuffed endotracheal tube (SCETT) group (*N* = 27) were compared in stable patients undergoing forceps and cryoprobe biopsy during bronchoscopy. The results suggest that UCETT under spontaneous respiration can provide satisfactory airway support and a shorter recovery time in stable patients.

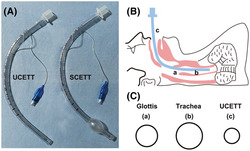

## 1 INTRODUCTION

Flexible bronchoscopy is the most frequently performed standard invasive procedure in the diagnosis and treatment of all kinds of lung diseases. Standard endotracheal intubation and rigid bronchoscopy are recommended to assist therapeutic bronchoscopy to maintain airways and hemodynamic stability.^1^, ^2^ General anaesthesia (GA) and neuromuscular blockade (NMB) are required for these patients, especially for those with multiple comorbidities and a potentially longer operation time.^3^, ^4^, ^5^ However, diagnostic bronchoscopy, including forceps and cryoprobe biopsy, is increasingly used in stable patients. More importantly, the durations of these procedures are often less than 1 h.^1^, ^6^, ^7^, ^8^ Therefore, whether a simpler and more practical endotracheal intubation is needed for a comparable airway support in stable patients during forceps and cryoprobe biopsy is still unclear.

The uncuffed endotracheal tube (UCETT) has great advantages in its ability to maintain spontaneous respiration (SP) without requiring use of mechanical ventilation, as compared to the standard cuffed endotracheal tube (SCETT).^9^, ^10^ A 5.0 mm inner endotracheal tube was successfully placed under thin bronchoscopic control, facilitating repeated insertion and removal of the bronchoscope under conscious sedation in stable patients with localized peripheral pulmonary lesions.^9^ These findings indicated that the UCETT was technically feasible. More importantly, no hypoxemia or laryngospasms were found in these studies.^10^ However, less studies used this simple method in forceps and cryoprobe biopsy for several reasons, such as lacking a proper UCETT compatible with widely used bronchoscopes. For example, the standard bronchoscope has an external diameter of about 4.9 mm, so to reduce resistance to gas flow through the tube, a compatible UCETT would have a diameter that is at least 6.9 mm or at least 2.0 mm wider than the diameter of the bronchoscope.^11^ However, presently, it is difficult to obtain an UCETT with an inner diameter (ID) of ≥7.0 mm.

Therefore, we recently modified a SCETT into a UCETT by removing the cuff,^12^ and we explored the efficiency and safety of the UCETT to assist biopsy in patients receiving forceps and cryoprobe biopsy. To our knowledge, no study has compared the use of adult UCETT‐assisted bronchoscopy to SCETT‐assisted bronchoscopy. In this study, we sought to evaluate the use of the UCETT during forceps and cryoprobe biopsy under SP by examining intubation, completion of bronchoscopy, hemodynamics, pulse oxygen saturation (SpO2) and complications.

## 2 MATERIALS AND METHODS

### 2.1 Study design

We make a detailed record of each patient undergoing both forceps and cryoprobe biopsy under bronchoscopic guidance in our own report form at the Beijing Anzhen Hospital between March 2021 and December 2021. This study was approved by our institutional review board (Ethics Committee Project No. 2022040X). Informed consent of bronchoscopy was obtained from all patients or their representatives before bronchoscopy. Medical records, operative reports and anaesthetic records were reviewed, and data regarding clinical indications for procedures, demographics, comorbid conditions, intraoperative hemodynamics and respiratory parameters were collected.

Eligible patients were 18 years or older and had stable baseline parameters, including percutaneous oxygen saturation (SpO2) > 95% in room air and hemodynamic stability at the beginning of the procedure.^13^ Participants had endobronchial lesions or peripheral localized solid lesions. Only standard forceps and cryoprobe biopsy were used during the procedure. Patients were excluded from the study if they suffered from coagulation disorders, were prescribed anticoagulation drugs, had uncontrolled cardiac arrhythmia or received bronchoscopy through tracheostomy or laryngeal mask airway. Other criteria for exclusion from the trial included incomplete data in medical files, central airway obstruction (CAO) and strong enhancement (≥40 Hu) in contrast‐enhanced CT images.^14^ All patients were assessed by the participating anaesthesiologists and pulmonologist, and all agreed to receive endotracheal intubation. GA and NMB were used to facilitate the bronchoscopic procedure if the patients suffered from refractory coughing and/or biting. We grouped patients according to the day of the week that the procedures were carried out. UCETT was performed on Monday and Wednesday, and SCETT were performed on Tuesday and Thursday.

Bronchoscopy was performed when adequate sedation (MOAA/S score, 3) was achieved.^15^ A 4.9‐mm bronchoscope (BF‐Q290; Olympus, Tokyo, Japan) with a 2.0‐mm working channel was advanced toward the target lesion through the bronchus. A 1.4‐mm radial endobronchial ultrasonic (rEBUS) probe (UM‐S20–17S; Olympus, Tokyo, Japan) was used to detect the target lesion through the working channel. After the target lesion was visualized by rEBUS, 1.8‐mm biopsy forceps (BF‐18, Micro Tech Co, Ltd, Nanjing, China) were advanced through the same route to the target lesion. Biopsies were performed until five visible specimens were obtained. Then, a flexible 90‐cm long, 1.9‐mm diameter cryoprobe (Erbokryo CA; ERBE, Tuebingen, Germany) was used. After the probe was placed against the targeted tissue, it was cooled for approximately 4–5 s. The frozen specimens attached to the probe tip was then removed and transferred gently to formalin for fixation. Three cryobiopsies were taken from each patient.^10^, ^14^ Electrocardiogram (ECG), non‐invasive blood pressure and pulse oximetry were monitored throughout the bronchoscopy and 15 min after the end of the bronchoscopy. A chest radiograph was performed to assess for pneumothorax within 3 h of the procedure.^16^

**FIGURE 1:**
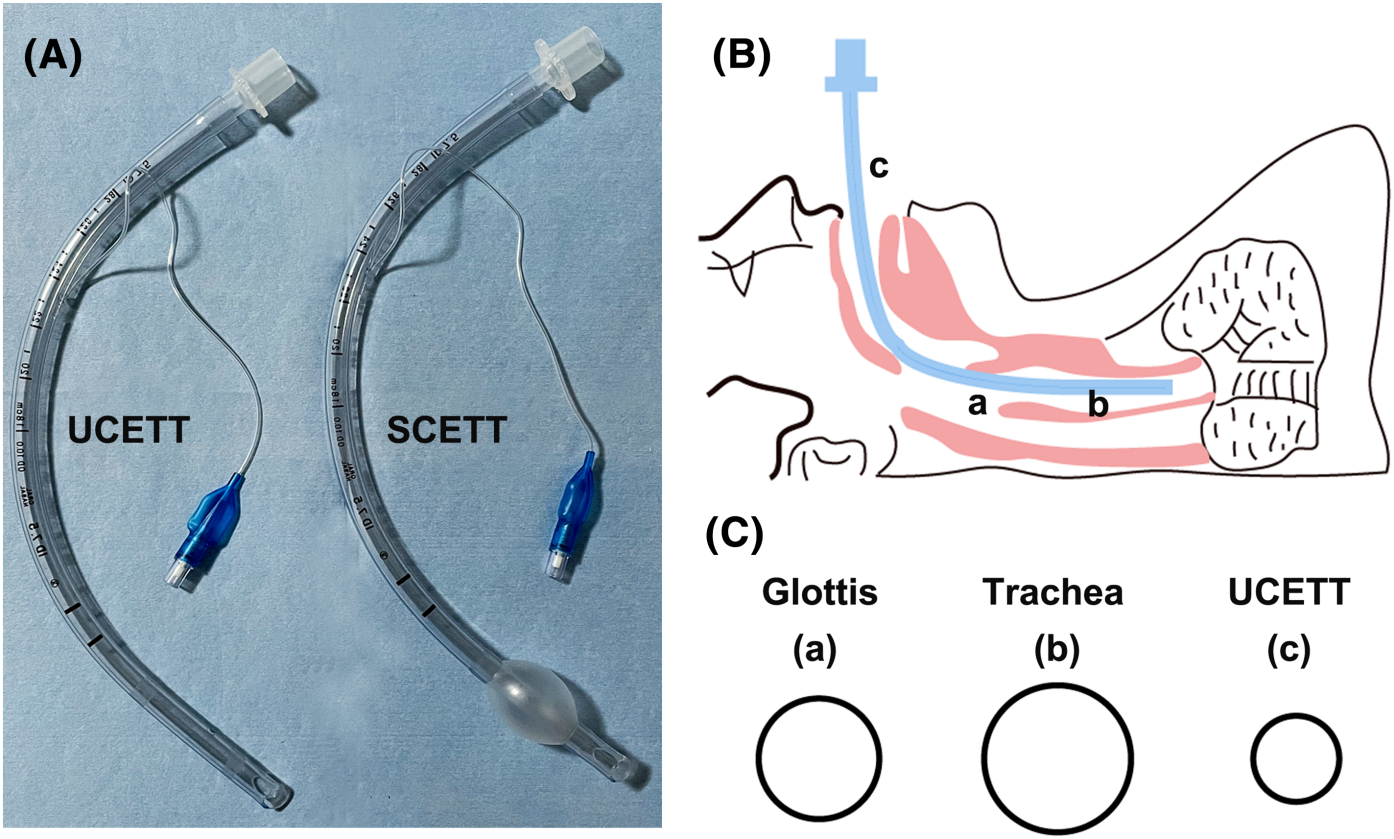
The schematic of modified adult UCETT and anatomical characteristics. (A) Adult UCETT of 7.5 mm is modified from the SCETT. (B) The schematic insertion of the UCETT into a patient. (C) The lumen surface of glottis, trachea and UCETT. The averaged dimensions at the level of the glottic (a) are approximately 12.6 mm in men and 10.3 mm in women, respectively. The tracheal dimensions (b) are approximately 15 mm in men and 11.6 mm in women. The diameter of UCETT (c) is approximately 7.5 mm.

The modified UCETT (7.5 mm ID) was achieved by removing the cuff of a 7.5 mm ID SCETT (COVIDIEN, Covidien IIc, USA) (Figure 1A). Patients were divided into the UCETT group and SCETT group according to different sedation protocols. Patients were closely monitored during the procedure by an anaesthesiologist, and the sedation protocol was also prescribed by the anaesthesiologist.

In brief, patients in the UCETT group received local laryngeal anaesthesia with 2% lidocaine, and then, propofol and remifentanil were administrated to maintain anaesthesia.^10^ The UCETT was inserted trans‐orally into the trachea under bronchoscopic guidance. All patients received supplemental nasal oxygen at 2–5 L/min. After verification of adequate mask ventilation, patients in the SCETT group received intravenous rocuronium. Two minutes later, a SCETT was inserted trans‐orally by bronchoscopic guidance and connected to an anaesthesia apparatus. Anaesthesia was maintained with propofol and remifentanil.^17^ A schematic of the location of UCETT and lumen surface of UCETT, glottic and trachea was shown in Figure 1B.

The primary technical outcome was to assess technical success including successful intubation and completion of bronchoscopy. The primary safety outcome was to assess the incidence of desaturation events. The secondary outcomes included the following: time to successful intubation, duration of bronchoscopy, recovery time, the proportion of patients with intraoperative hypertension or hypotension, cardiac arrhythmias and other complications.

All patients were graded by Mallampati Classification prior to bronchoscopy.^18^ Time to successful intubation was defined as the time elapsed from the bronchoscope entering the mouth to departing the mouth after location of the tube was confirmed. Duration of bronchoscopy was calculated from the administration of sedation until the flexible bronchoscope was removed from the tracheobronchial tree.^1^ Incidence of desaturation events was defined as Spo2 < 90% when intraoperative management was stably maintained.^4^ CAO was defined as occlusion of ⩾50% of the trachea, main‐stem bronchi, bronchus intermedius or a lobar bronchus.^19^ GA was defined as intravenous anaesthetics via continuous infusion or intermittent injection with the use of rocuronium. Rocuronium was one of the most common used NMB 4. Recovery time was defined as the time from end of bronchoscopy to full alertness. Full alertness was defined as the first of three consecutive modified observer's assessment of alert/sedation (MOAA/S) measurements out of five after the end of the procedure.^20^ The severity of intra‐procedural bleeding was defined as minor (continued suctioning of blood from the airways; bleeding stops spontaneously), moderate (intubation of the biopsied segment with the bronchoscope in the wedge position; use of adrenaline or cold saline stopped bleeding) or severe (placement of bronchus blocker or catheter, or application of fibrin sealant resuscitation; blood transfusion, admission to critical care unit or death).^21^ Hypertension was defined as systolic blood pressure >140 mmHg. Hypotension was defined as systolic blood pressure < 100 mmHg. Cardiac arrhythmias were classified as major or minor according to Shrader and Lakshminarayan's classification scheme. Major arrhythmias were those having the potential to affect hemodynamic stability and included sinus bradycardia (<40 bpm), supraventricular tachycardia (>120 bpm), premature ventricular contractions (>50/h) and any other ventricular arrhythmias. All other arrhythmias (e.g., sinus tachycardia, premature atrial contractions and atrial fibrillations) were considered minor arrhythmias. Sinus tachycardia was defined as heart rate >100 bpm.^22^

### 2.2 Data analysis

Statistical analysis was performed using SPSS 20.0 software (SSPS Inc., Chicago, IL, USA). Continuous variables are presented as mean ± standard deviation (SD). Categorical variables are presented as numbers and percentages. The relationship between categorical variables was assessed using the Chi‐square test. The connection between quantitative variables was evaluated using independent‐sample *t* test. Statistical significance was set at *p* < 0.05.

## 3 RESULTS

**FIGURE 2:**
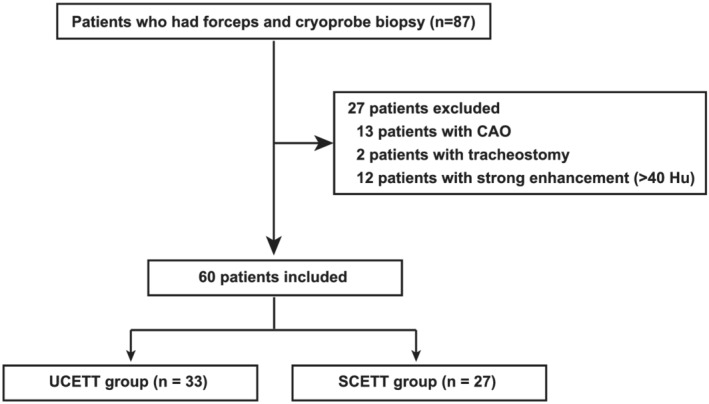
Flow chart of patient enrolment

A total of 87 patients who had forceps and cryoprobe biopsy were collected, and 60 patients were enrolled in this study with a mean age of 59 ± 13 years. Patients who suffered from CAO, those who underwent bronchoscopy through tracheostomy and those who had lesions with strong enhancement (≥40 Hu) were excluded. The flow chart of patient enrolment was shown in Figure 2.

There were 27 patients in the SCETT group and 33 patients in the UCETT group. The distribution of endobronchial lesions, mostly in the main bronchus and lobar bronchus, was similar in both groups. The mean lesion size, defined according to the largest diameter on CT scans, was 26 ± 0.7 mm in the SCETT group and 25 ± 0.7 mm in the UCETT group (*P* > 0.05). Baseline characteristics were similar in the two study groups, as shown in Table 1.

**TABLE 1:** Demographic and clinical characteristics (*n* = 60)

Characteristics	SCETT group(*n* = 27)	UCETT group(*n* = 33)	*P*
Sex			
Male	16	22	0.599
Female	11	11	
Age (years)	57.2 ± 13.4	59.8 ± 12.6	0.538
Smoking			
Yes	16	17	0.519
No	11	16	
Height (cm)	168.2 ± 6.7	169.9 ± 9.0	0.286
Weight (Kg)	66.9 ± 7.0	68.9 ± 9.3	0.165
BMI (Kg/m2)	23.6 ± 1.5	23.7 ± 2.0	0.718
Mallampati Classification	1.3 ± 0.6	1.2 ± 0.5	0.226
Chronic bronchitis			
Yes	11	14	0.299
No	16	19	
Hytertension			
Yes	10	11	0.765
No	17	22	
Coronary heart disease			
Yes	12	13	
No	15	20	0.693
Baseline parameters			
SPO2 (%)	99.1 ± 1.1	99.0 ± 1.1	0.965
PCO2 (mmHg)	39.9 ± 3.2	40.7 ± 2.8	0.617
SBP (mmHg)	125.6 ± 10.7	126.3 ± 10.2	0.522
DBP (mmHg)	70.6 ± 8.7	71.1 ± 8.6	0.755
HR (bpm)	74.1 ± 8.2	77.1 ± 8.6	0.844
Characteristics of lesions			
Endobronchial lesions	11	12	0.729
Left main bronchus	2	3	0.814
Left lobar bronchus	5	3	0.285
Right main bronchus	1	0	0.265
Right lobar bronchus	3	6	0.728
Peripheral lesions	16	21	0.729
Left upper lobe	2	3	0.814
Left lingual lobe	1	2	0.677
Left lower lobe	2	4	0.545
Right upper lobe	2	5	0.353
Right middle lobe	6	4	0.256
Right lower lobe	3	3	0.295

UCETTs and SCETTs were successfully inserted, and bronchoscopic procedures were performed on all 60 patients. Therefore, the technical success rates were 100% in both groups. Only 9.1% (3/33) of the patients in the UCETT group exhibited a drop of SPO_2_ < 90% during the bronchoscopy, compared to 7.4% (2/27) of the patients in the SCETT group (*P* = 0.545). The SPO_2_ returned to normal levels (SPO_2_ > 95%) soon after the bronchoscopy was withdrawn in these patients. The mean SpO_2_ levels at 10, 20, 30 and 40 min after the procedure and post‐bronchoscopy were not significantly different between the two groups, as shown in Table 2.

**TABLE 2:** Monitoring of SpO2 during bronchoscopy

Time	*n*	SCETT group	n	UCETT group	*P*
10 min	27	97.7 ± 2.1	33	97.8 ± 1.8	0.293
20 min	27	97.1 ± 1.8	33	97.4 ± 1.8	0.944
30 min	24	97.5 ± 1.2	32	96.9 ± 1.6	0.120
40 min	7	98.1 ± 1.1	8	96.3 ± 1.0	0.909
Post‐bronchoscopy	27	99.4 ± 0.7	33	99.2 ± 0.9	0.140

It took 96.7 ± 11.6 s to accomplish intubation in the UCETT group, compared to 84.5 ± 4.8 s in the SCETT group (*P* < 0.001). The durations of bronchoscopy were similar between the two groups: 37.2 ± 5.6 min in the UCETT group and 35.9 ± 6.8 min in the SCETT group (*P* = 0.814). The durations of procedure were ≤40 min in most cases (45/60, 75%). Patients recovered faster in the UCETT group (6.0 ± 0.9 min) than those in the SCETT group (11.6 ± 1.8 min) (*P* = 0.001).

Major bleeding, laryngospasm and major arrhythmias did not occur in either group. Intraoperative hypertension was seen in 18.2% (6/33) of the patients in the UCETT group and 11.1% (3/27) of the patients in the SCETT group (*X* ^2^ = 0.582, *P* = 0.445). Intraoperative hypotension occurred in 12.1% (4/33) of the patients in the UCETT group and in 33.3% (9/27) of the patients in the SCETT group (*X* ^2^ = 3.937, *P* = 0.047). Sinus tachycardia occurred in 15.2% (5/33) of the patients in the UCETT group and in 7.4% (2/27) of the SCETT group (*X* ^2^ = 0.864, *P* = 0.353). Premature atrial contractions occurred in 9.1% (3/33) of the patients in the UCETT group and 11.1% (3/27) in the SCETT group (*X* ^2^ = 0.067, *P* = 0.795). These changes rapidly returned to normal levels once the bronchoscope was withdrawn. There was no significant difference in mean PCO_2_ levels after bronchoscopy between the UCETT and SCETT group (39.9 ± 6.3 mmHg vs. 40.5 ± 2.0 mmHg, *P* = 0.238). All patients suffered from different degrees of throat pain in both groups. Nausea only occurred in 15.2% (5/33) of the patients in the UCETT group, as compared to 40.7% (11/27) of the patients in the SCETT group (*P* = 0.026). There was no statistical difference between the two groups in vomiting incidence. Minor and moderate bleeding occurred in 45.5% (15/33) of the patients in the UCETT group, as compared to 48.1% (13/27) in the SCETT group (*P* = 0.835).

## 4 DISCUSSION

In this trial of forceps and cryoprobe biopsy in stable patients, the use of UCETT resulted in a similar technical success and a shorter recovery time compared to the use of SCETT. However, these findings should be interpreted as preliminary and require replication in prospective trials.

The present study evaluated the efficiency of adult UCETT for forceps and cryoprobe biopsy in stable patients. These results showed that adult UCETT use had a high technical success rate similar to that of SCETT use. Previous studies described the use of paediatric UCETT in bronchoscopic forceps biopsy, which facilitated repeated insertion and removal of the bronchoscope.^9^, ^10^ However, forceps and cryoprobe biopsy have been increasingly used in interventional bronchoscopy.^23^, ^24^, ^25^, ^26^ A relatively larger UCETT is needed for cryoprobe biopsy. In this study, UCETT under SP provided a reliable airway for cryoprobe biopsy, because both the cryoprobe and bronchoscope can be retracted together during procedure. In addition, UCETT under SP did not increase the incidence of desaturation. Thus, our strategy provides an easier procedure and lessened interruptions for oxygen enrichment due to the combination of forceps and cryoprobe biopsy under SP.

The safety of UCETT in patients with SP was another vital aspect. Cardiovascular complications, including unexpected increase or decrease in SBP and HR, may occur during bronchoscopy because of abnormal autonomic nervous activity.^27^, ^28^ In our study, the proportion of hypertension and minor arrhythmias was similar between the two groups. Notably, the proportion of hypotension was less in UCETT group as compared to that of the SCETT group. These results may be associated with different depth of sedation and less usage of sedative drugs during bronchoscopy.^28^ Another advantage was that patients in the UCETT group breathed spontaneously without mechanical ventilation. Therefore, patients in UCETT group recovered quickly and demonstrated a lesser frequency of nausea when compared with patients in the SCETT group. More importantly, adverse events related to the usage of NMB, including decreased airway tone, impaired oxygenation and impaired respiratory effort during recovery from anaesthesia, were not found in this study. In addition, when patients readily regain cognitive function after bronchoscopy, physician‐patient communication may be enhanced.^20^

This study had several limitations. First, it was a small, retrospective report with a single aim. Second, only stable patients with endobronchial lesions or peripheral localized solid lesions were included. Third, patients were excluded from this study when they had diffuse interstitial lung disease, central airway obstruction or strong enhanced lesions. We are planning to perform a prospective randomized trial in the future, and this study will include patients' feedback about their experience of the procedure.

Among stable patients receiving forceps and cryoprobe biopsy, the use of UCETT under SP provides not only a satisfactory airway support and a shorter recovery time but also a stable oxygen saturation during the procedure. Thus, in stable patients, the UCETT may be an option to assist forceps and cryoprobe biopsy.

## CONFLICT OF INTEREST

The authors have no conflict of interest to declare.

## ETHICS STATEMENT

This study (2022040X) was approved by the Institutional Review Board at Beijing Anzhen hospital. A waiver of consent was obtained due to the retrospective nature of this study.

## AUTHOR CONTRIBUTIONS

MT was involved in conception and design, collection and assembly of data, data analysis and interpretation, and manuscript writing. XXW, QS and HL were involved in collection and assembly of data and review. FGZ and HZ approved the final version of the manuscript.

## Data Availability

All data included in this study are available up on request by contact with the corresponding author.
